# Logic and Psychology
*Prolegomena Logica: An Inquiry into the Psychological Character of Logical Processes. By Henry Longueville Mansel, M.A., Fellow and Tutor of St. John's College, Oxford. Whittaker and Co.


**Published:** 1854-01-01

**Authors:** 


### Art. V.—LOGIC AND PSYCHOLOGY.*

Though logic has been cultivated as a science from the time of the Greek civilization, and maintained its place in the curriculum of learning, when the human mind was making but little progress in branches of knowledge which have been matured in after ages, nothing can be more vague, as our author complains, than the manner in which

* Prolegomena Logica: An Inquiry into the Psychological Character of Logical Processes. By Henry Longueville Mansel, M.A., Fellow and Tutor of St. John's College, Oxford. Whittaker and Co.

logic has often been treated, even in recent times. The fact is, that the subject has been looked at from so many different points of view, and has been so mixed up with matters not strictly belonging to it, that any attempt which may tend, on a philosophical basis, to promote its elimination from extra-logical incumbrances, and to place it on a more satisfactory footing, may be regarded as a benefit to science. Such an attempt is the work before us; and whatever has been the result, we can assure our readers that the book is written with no ordinary ability for such discussions as it contains.

The author's design is to exhibit more fully the relations existing between logic and psychology. The work is not an introduction to logic; indeed, it presupposes in its readers some idea of the subject, beforehand. It aims at what is, in the order of nature, prior to logic, though of later scientific development—an inquiry into the constitution and laws of the faculty of thinking, as these laws are necessarily assumed by the logician. Many German writers have elaborated formal logic, but their labours are little known in this country; and the nature and scope of the science are often but loosely apprehended, in consequence of the failure of regarding it sufficiently in a psychological point of view. To this failure, our author attributes largely the confusion of systems which the very term *logic* still calls up in the minds of those who are in any degree imbued with the literature of the subject.

"We have recently passed a statute [at Oxford] enacting that a proficiency in logic is to have considerable weight in the distribution of honours. But the present state of logical literature is not such that the mere mention of the subject is sufficient. What logic does our statute recommend? Is it Aristotle?—is it the Schoolmen?—is it Bacon?—is it Aldrich?—is it Archbishop Whately?—is it Mr. Mill?—is it Mr. De Morgan?—is it Wolf?—is it Kant?—is it Hegel? Most of these already exercise some indirect influence on our studies and examinations, and it is merely the want of good translations that saves us from being overwhelmed by an additional mass of incongruities from Germany."

Mr. Mansel insists, that the only way in which the formal view of the science, which has been gradually advancing to perfection since the days of Kant, can be rendered thoroughly complete, is by a more definite study and appreciation of it in connexion with its undoubted psychological basis.

Hence the work before us consists of a series of psychological criticisms, mainly limited to logical questions, but many of them, nevertheless, of high interest to the student of general psychology; for the author has discussed the various points which he has selected, with an acuteness and analytical power which must at once rank him with the ablest writers on the subject, whether we follow him or not in all his theories and conclusions. It is evident that he has well studied the Kantian speculations, and that he has surveyed them with an intelligence too great to allow him to be either a blind partisan or an indiscriminate opponent. From having ourselves, in an humble way, for some time past, officially attempted something towards introducing the student of general psychology and logic to an historical acquaintance with the main doctrines of Kant, we were glad to find our private estimate of this greatest of the German metaphysicians so much corroborated by the opinion of our author; we say *greatest*, not by way of pronouncing on the degree of genius or invention which Kant may be supposed to have possessed, in comparison with a few other men who are accounted stars of the first magnitude, by their countrymen, in the hemisphere of philosophy,—but simply because Kant knew better *where to stop* than either Leibnitz, or Fichte, or Schelling, or Hegel; not to add that the whole school which came after him owes to him its impulse, far as it deviated from his track.

"The reader who is familiar with Kant's writings will probably discern obligations to the Critical philosophy in almost every page, even where the language of Kant has been departed from, and the difference in detail is such as would not justify a direct reference to his works. The method and material for thinking derived from the study of the Kantian philosophy, is in many respects far more valuable than the direct information communicated. This is the case especially with a student who views that philosophy from the psychological, rather than the metaphysical side, in its relation to Hume and Locke, rather than to Wolf and Leibnitz; and who endeavours to combine the materials thence obtained with the most valuable results of the Scottish philosophy, which owes its rise, like the Kantian, to the scepticism of Hume."—*Preface.*

As compared with Kant's view of logic, our author's (which aims at being very strictly formal, and not applied or mixed in any degree) would base itself on more limited notions of the provinces of understanding and reason than even those of Kant himself. From the view taken of the office of logic in the Eclecticism of Cousin, of course he widely differs; for, as he justly observes, this system "approaches, in aim at least, if not in method, nearer to the philosophy of Schelling and Hegel, than to that of Kant." Yet, with a candour which reigns through all his pages, and which is studiously desirous of giving to every one his full due, our author is liberal in his acknowledgments of the general benefit which he has derived from the writings of M. Cousin. In the same spirit Mr. Mansel expresses that he is much indebted to the writings of Sir William Hamilton, though by no means uniformly agreeing in his opinions. In closing the general statements of his preface, our author, for the sake of clearness, pleads for some latitude in affixing meanings to words—we mean the right to adopt, if necessary, new terms, and to put a new interpretation on old ones, according to the Aristotelian maxim—*ὀνοματοποιεῖν σαφηνείας ἕνεκεν.*

The author, in accordance with the views of Kant, treats logic as the "Science of the Laws of Formal Thinking." Hence the necessity of inquiring into the actual nature of thought as a mental operation, its laws, and the extent of its efficient application. "With these alone, and with no other possible psychological functions, has the logician to do." He must distinguish between Thought proper, and all other mental phenomena, if he wishes for a real basis for his science. Two elements at least are implied in every state of consciousness; "a conscious subject, and an object of which he is conscious." This object may or may not, in any given case, involve the existence of an external world, such as is commonly understood. The object may be distinct from *self*, or it may be a modification of ourselves—that is, of our own minds. All that our author here contends for is, that there is present in the act of thought such an individual object, "whether thing, act, or state of mind," and that we are conscious of such object (thus interpreted) as existing within or without ourselves. Hence whether, with Sir W. Hamilton, we admit an ontological ("natural") dualism or not, at all events, a psychological dualism is implied in the very notion of consciousness. We suppose that the author would regard his theory as not more independent of all the forms of the Peripatetic idealism than of the Berkleian, or the modern German—contending only that, in thought, we are *conscious*, and conscious of *something*, be it what it may. This *something* is simply to be distinguished from our consciousness of it.

But, in thought, there is more than the above. A being is conceivable whose successive states of consciousness might leave no trace or memory of them behind: he would then have no knowledge of such objects "as referred to separate notions." He could not say, "What I see is a man, or a horse." He would be wanting in the distinctive feature of thought—namely, the concept or general notion resulting from the comparison of objects. Hence the importance of distinguishing *intuitions* from *thoughts*. The term intuition is used by Mr. Mansel in the German sense (*anschauung*), so as to include all that arises in connexion with the perceptive faculties (whether of external or internal objects) or from imagination—to include every act of consciousness which has for its immediate object any individual thing, state, or act of mind, presented under the condition of separate existence, either in space or time, or both. In *intuitions*, such objects, or *quasi* objects, are immediately related to the conscious mind: in *thoughts*, the object is mediately related to it through a concept gained by comparison. The act of thought and its object are here distinguished, as in the *νόησις* and *νόημα* of the Greeks, the former answering to our term conception, and expressing a mental act—the latter to the term concept, of established usage in the French Eclectic school, and now much used among ourselves as a convenient abbreviation, expressing the object of the act. Sir W. Hamilton, whose theory is the same, in like manner distinguishes "an absolute or irrespective (*immediate*) cognition," from the "mere relative and mediate knowledge which subsequently we compass of the same object, when, by a comparative act of the understanding, we attach it to a class, that is, think or recognise it by relation to other things, under a certain notion or general term." An intuition thus contains but two elements—subject and object, in present relation to each other: a thought has three elements—the thinking subject, the object thought on, and the concept as a medium to both. In mere sensible intuition, the senses present no distinctions —a landscape is only to them a single object whose parts are confused: to distinguish trees, fields, houses, &c., requires a comparison and classification of the parts relatively to so many separate concepts existing in the mind; and such classification is an act of thought; so that thought exists whenever the senses present objects to us, in ordinary, with our faculties awake and attentive. When I say—That is a tree, that is a flower, there is both intuition and thought.

Hence a further distinction : all intuition is direct and presentative —all thought indirect and representative. For in every act of consciousness, the ultimate object is an individual; and in intuition the object is presented to the mind directly and *per se*, irrespectively of anything but itself: in thought, the individual is presented by means of a concept which contains attributes also belonging to other like individuals. The concept, therefore, is a general notion, the result of prior presentations. The isosceles triangle drawn on the paper before me, and which is pictured on my retina, is an intuition; my notion of it as triangle merely, having the common properties, is a concept.

Mr. Mansel fully adopts Sir W. Hamilton's doctrine of perception, which discards every modification of the theory that our cognisance of external objects, as such, is the mere result of a representation of them in consciousness—and maintains that we are *conscious* not only of our own (subjective) sensations, which none dispute, but that we are also *conscious* of the external object. The question, of course, here is, whether (be the external object what it may in itself) we can be said to be conscious of it, in the same sense in which we are conscious of a certain change in our mental state? Is my knowledge of this change in myself the same kind of knowledge which I have of the supposed cause of that change? This point appears to us not to be sufficiently cleared up in the acute speculations of Sir W. Hamilton, nor in those of the author.

Perception (sensitive) is, according to him, synonymous with intuition of external things—internal intuition relating to mental states or acts, as objects taken cognisance of as being in the mind. Yet he admits that the usual exercise of the senses on objects does not consist of mere intuition, but is accompanied by an act of thought. According to this, perception would seem to include the latter, which, however, is contrary to the author's theory, which limits perception expressly to what is presentative only, whereas thought is representative. Again, he defines imagination, as a product, to be the "consciousness of an image in the mind resembling and representing an object of intuition, and being both presentative and representative." But is there, then, objectively, any difference, according to the author's own previous theory, between the intuition of an external object and a product of imagination as above described, excepting that the former is a case of "external," and the latter of "internal" intuition. We think the author should have somewhat more elaborated his doctrine, or at least his language, regarding perception, and have more sharply distinguished the cases to which he would apply the terms intuition, perception, thought, and imagination respectively. For, while he expressly limits perception to intuition, or to presentative consciousness, he speaks, nevertheless, of the "exercise of the senses upon present objects" (sensitive perception, we suppose) as "not *consisting* of mere intuition," and of imagination as being both presentative and representative to consciousness; while he at the same time characterizes representative power as a function of thought only, though it is not easy to see wherein an image raised in the fancy differs in the consciousness of the moment from the intuition of external perception, except in being "internal." Even granting that an object of external intuition were always consciously represented, when there is an image in the fancy, which we doubt, this representation is not of the kind which our author claims in concepts—namely, a general notion gained by comparison and classification, or what he terms an act of thought. Our space will not allow us to more than indicate the above confusions, or at least difficulties. We must go on to remark that, after having described thoughts as those states of consciousness in which the object is mediately related to the mind through concepts gained by comparison, and which are not capable themselves of being depicted to sense or imagination, though their ultimate objects are individuals, our author gives, as a second characteristic of all concepts, and illustrative of the first, that they require to be fixed in a representative sign. This is exemplified in Kant's refutation of Leibnitz's principle of *identitas indiscernibilium*, which is applicable, says Mr. Mansel, to concepts, but not to objects of intuition (query external?). Well and good, if we leave time as well as space out of the account. No doubt, however much alike two outward objects are, they can never be identical, because they occupy different spaces ; " whereas my notion of hunger, or fear, or volition, is a general concept, having no relation to one period of time rather than to another ; and as such, like other concepts, requires a representative sign." There is, however, a sense not here noticed, in which Kant might have extended his refutation of Leibnitz's theory, by putting concepts on a par with external objects of intuition : for as a concept is not always actually thought, in the mind, but only on occasion, all our concepts may be regarded as so far numerically distinct, as much as the spacial intuitions of sense, since my concept of triangle while I now write is not the same, in point of *time*, as the concept of yesterday, though it may be the same in all other respects.

In pursuing the characteristics of thought, our author holds that language is indispensable even to its formation. On this principle Condillac denied that brutes have any memory, since they have not language. Mr. Mansel would say that memory is not thought, while reminiscence is ; and he agrees with Aristotle, that memory is common to man and brutes, but reminiscence peculiar to the former. We can only state, on this subject, that, while fully admitting the reality of the distinction of Aristotle between μνήμη and ἀνάμνησις, we have always strongly doubted whether what is often called memory in brutes, ought not rather to be regarded as mere association. With respect to the relation of language to our psychological acts, we do not doubt that without the former our range of knowledge would be extremely limited, and that our first infantile knowledge is of individuals whose names we are taught. Nevertheless, language must always, even in its most abstract terms, be, like all other symbols, a sign of *something*. We could never, without an elaborate system of these symbols, have a differential calculus or an algebra, for memory could never build up or retain the high generalizations of these sciences, apart from such marks or signs—yet they must be signs of something, and that something can be nothing but ideas or concepts, agreeably to Mr. Mansel's definition, that thought is the " act of knowing or judging by means of concepts."

Our author's Eclectic Kantism carries him on to another point most important, which is an inquiry into the limits of thought ; and he decides, with the great German metaphysician, that it is " only operative within the field of a possible experience ; that is, upon such subjects as can be presented in an actual intuition, or represented in an imaginary one." We regard this principle as deserving of the careful attention of all who would desire to have just ideas of what true knowledge is, as distinct from vague and indefinable ideas. The concept plane-triangle as a right-lined figure of three sides, does not in itself contain those attributes which make any species equilateral, scalene, or right-angled, but it is capable of being combined with any one of the three, either in an actual external intuition, or in an imaginary figure. On the other hand, a right-lined figure of two sides is no concept at all, for it cannot be individualized. Hence the criterion of positive thinking, as distinguished from certain words which are in fact only a negation of thought. We must not mistake words which are in themselves intelligible, for concepts that may be formed and reduced to an actual intuition in sense or imagination. We can understand the meaning of the three words *right-angled equilateral triangle*, but the object is inconceivable. This doctrine is undeniable. With regard to the mental operation called *abstraction*, we agree with the author in getting rid of the objection of Berkeley and Hume to the abstract notion—say of a triangle—namely, that our actual idea (concept) of a triangle must necessarily be particular, and cannot be general; for, as Mr. Mansel remarks, we do in the majority of cases employ concepts (or general notions) as instruments of thought, without actually submitting them to their always possible test of individualization. We can judge and reason about triangle in general, without trying to imagine any particular one. On this account we think that Dr. Thomas Brown's designation of this fact, as an example of "*relationism*," is not amiss—a term which he uses to express that it is simply a certain relation in which all the individuals agree that is the true object of thought in these cases. This view of the matter gets rid of all the difficulties and absurdities of other theories which have prevailed, such as realism, normalism, and conceptualism. Drobisch has observed that abstraction may be viewed either as psychological or logical. We can psychologically fix our minds on some one property common to many individuals, that is, we can abstract in this sense; but we cannot assign incompatible predicates to the same subject as we should do if we tried to think of a triangle that was neither isosceles, nor scalene, nor equilateral, or one that was all three at once. This would be a logical contradiction. So far, and so far only, Berkeley was right.

As thought involves the possible application of concepts, as before explained, to individuals, either as objects of external or of internal perception, our author proceeds further to remark that the "possibility of any branch of scientific inquiry depends on the psychological question—*how many presentative faculties has man?* since every such faculty may furnish distinct materials for thought." The only objects of science are those which can be in any way presented as objects of an immediate intuition, external or internal. Thus, physical science presents us with material phenomena; moral science presents the inward fact of moral self-approval and remorse on account of an action for its own sake; æsthetics is a possible branch of inquiry, because we have certain emotions on contemplating the works of nature and art.

Our author justly intimates that the psychological distinction which he adopts between what is presented in intuition and what is represented in thought (concept), is so far from original in his own pages that it has been repeatedly marked with greater or less distinctness by modern philosophers. Locke, in the second book of his essay, maintains that it is not in the power of man, by any means, "to invent or frame one new simple idea in the mind;" he can only compound or divide the materials given to him. It has been so much the fashion to disparage our great English metaphysician, throughout, on the Continent, and sometimes at home, and to hold him to a precision of language which, in his day, modern speculative philosophy had still to attain; that we are glad to find the author doing him candid justice, in allowing that his *ideas of sensation* and *ideas of reflection* point correctly enough to the two great sources of external and internal intuition; although it may be conceded that his choice of terms here is not happy, nor always consistent, and that reflection (his second source of knowledge) can only be understood in an improper sense, as being synonymous with consciousness of what is passing within us, as Dugald Stewart accurately remarked. Hume pointed to the same distinction in the sources of our knowledge, terming them, with too little accuracy of explanation, "impressions and ideas;" for he made no other difference between the two than that which lies in their respective degrees of vivacity; on which doctrine Reid smartly remarks, that "it will follow that the idea of a lion is a lion of less strength and vivacity; and here arises a question, whether the idea of a lion may not tear in pieces and devour the ideas of sheep, oxen, horses, men, women, and children."

Mr. Mansel, as may be gathered from what has preceded, entirely agrees with Kant in the result of his attempt to disentangle the confusion which prevailed, before he wrote, respecting *ideas;* and he considers it to be one of the most valuable principles of the Critical philosophy that the understanding has *no power of intuition;* or, as our author explains it, that the act of thought cannot create its own object. Now, we quite agree with Mr. Mansel that thought, as described by him to be mediate and representative, and requiring to be based on an "immediate and presentative fact of consciousness," cannot create its own object. It is impossible for a man born stone blind, for instance, to imagine the general concept *colour*, for he is utterly unable to refer it to any individual example, as blue, red, or other. Nevertheless, we object to Kant's general doctrine of intuition, because he limits it to sensibility (*sinnlichkeit*), under which he places not only our cognizance of external objects, but even of all the internal modifications of the conscious *ego*. For instance, our notion of time is, according to him, a form of internal sense—a doctrine which has been regarded as one of the most fundamental errors of his system, even by many of Kant's most candid critics, who have justly maintained that such an idea or notion as *time* is exclusively an affair of the understanding, which alone (and not sense) can take cognizance of it.

It is further remarked that, as our knowledge must flow entirely from what our faculties, sensuous or others, can *present* to us, we have hence some light thrown on the distinction between *positive* and *negative* ideas. A positive intuition (a sound, for instance) is one that has been presented to us in actual consciousness; a positive concept (e. g., quadrilateral, apart from its species) is one formed from such presentations. A negative intuition is one that has never been so presented—a negative concept is no concept at all. If I had only seen a red colour, I should have a positive idea (intuition) of it, but only a negative one of blue. I have a positive concept of quadrilateral figure—a negative concept (that is, none at all) of a figure with two sides only. With more candour towards Locke than most who are imbued with the Kantian and the Eclectic metaphysics, our author adds:

"When Locke declared infinite space and infinite duration to be negative ideas, he was right, if we grant his hypothesis of their origin. The former he derived from sensation; and all the space we can actually perceive by the senses is finite. The latter he derived from reflection; and every duration which we have personally experienced, is finite also."

We have always regarded it as an error in the French Eclectic school to maintain, as M. Cousin does, that our psychological idea of infinity is positive. We may admit, that so far as we can carry our idea *towards* infinity, it *is* positive: but this does not satisfy the case. No assignable magnitude fulfils the condition—what the mind aims at is something always greater still.

Among our author's criticisms, is one on the disputed subject of logical definition. The scholastic logicians defined by genus and differentia, so that nothing was definable that could not be regarded as species (e. g., man is a rational animal). Descartes and Locke, on the other hand, rejected this restriction, and maintained that it is only the *simple idea* that cannot be defined.

"Both are right, according to their different meanings of definition. With the former, it signifies the resolution of a complex *general concept* into the simpler concepts which it comprehends: with the latter, it is the resolution of a complex *individual object of sense* into the simpler concepts of which it is composed. No definition, as Locke truly observes, will convey the idea of whiteness to a blind man. But no definition (in the scholastic sense) was ever intended to accomplish this object. Concepts as such are not capable of being presented in sense or imagination. If the purpose of logical definition were to enable us to form an *idea*—i. e., a representative image of an object—pointing it out with the finger would be a far more satisfactory definition than any verbal analysis. But ideas, in this sense, have no connexion with logical definition. Locke's ideas of sensation, simple or complex, are all excluded from the province of definition as being individuals—i. e., as not being concepts at all. An example adduced by Descartes, Locke, and Leibnitz, will illustrate the distinction more clearly. The concept of a chiliagon is a regular polygon of 1000 sides. As addressed to the sense, this definition would not enable any man to distinguish an individual figure of the kind by sight from another which had 999 sides; but, as addressed to the understanding, it is sufficient for the demonstration of the mathematical properties of the figure."

Mr. Mansel employs the same distinction (which is certainly an important one) as a ground of criticizing some of the modes used for logical notation. Logic is concerned with thought, and thought, in the strict sense, is wholly concerned with "concepts." On this account, he objects to the representation of the relation of terms in a syllogism by that of figures in a diagram. To do so he regards as losing sight of the distinctive mark of a concept—that it cannot be presented to sense; and as confusing the mental inclusion of notions in other notions with the inclusion of dimension within dimension. Hegel is of the same opinion, pronouncing it useless to attempt to represent conceptions by spacial figures and algebraic symbols. Our author instances the diagrams of geometry as furnishing no suitable precedent for such a method, "for they do not illustrate the *form* of the thought, but the *matter*,—not the general character of the demonstration as a reasoning process, but its special application as a reasoning about magnitudes in space." With all deference to the authority of a writer of such merit as our author, we demur to his objections. Taking the term concept in the author's sense, it must be allowed that every concept has a limit, that two concepts may wholly exclude each other, may exclude each other partially, may be identical, or that one may contain the other. An example of the first would be *tree* and *moral-being*, of the second *mathematician* and *linguist*, of the third *man* and *rational-animal*, of the fourth *conic-section* and *ellipse*. Now, why may not these relations be illustrated by geometrical figures? As to algebraic symbols, which were adopted by Aristotle himself, whenever he employed the first three letters of the Greek alphabet as terms, they are eminently suited for the purpose by their brevity; and of the power of an algebraical calculus to express complex propositions, and thus to extend the development of Logic, and to give precision to its notation, we have a recent example in Professor De Morgan's "Formal Logic."

We have dwelt so long on our author's most elementary principles, that we have not much space left for their application. He considers that the three usual divisions, apprehension (conception), judgment (proposition), and reasoning (syllogism), rightly express distinct classes of mental operations, though they all point to one single psychological function as their source—namely, *thought*. He identifies every act of consciousness, in a certain sense, with *judgment*, there being always a conviction of the presence of the object of such act, either externally in space, or internally in the mind—a conviction amounting virtually to the proposition, "This is here." Thus every operation of thought, even the single concept, is a judgment, psychologically considered, though not logically; for in the latter case we must have two objects of thought, and the logical judgment expresses their relation. Reasoning is the most complex of the three operations, as in it two concepts are determined to be, in a certain manner, related to each other, through the medium of their mutual relations to a third.

It may have occurred to our logical readers to ask how the author's theory of concepts can be made to square with *singular* propositions, having the force of universals, as is commonly allowed by logicians, since the predicate is said of the whole of the subject-term? We think his remarks on this part are somewhat far-fetched, and they show that the terms of a proposition are not always concepts, in Mr. Mansel's sense of the word, as above explained. His reply to the question is as follows. If I say—

"Cæsar was the conqueror of Pompey, the immediate object of my thought is not Cæsar as an individual existing 2000 years ago, but a concept now present in my mind, comprising certain attributes which I believe to have co-existed in a certain man. I may *historically* know that these attributes existed in one individual only; and hence my concept, virtually universal, is actually singular, from the accident of its being predicable of that individual only. But there is no *logical* objection to the theory that the whole history of mankind may be repeated at recurring intervals, and that the name and actions of Cæsar may be successively found in various individuals at corresponding periods of every cycle."

We confess that this does appear to us very much indeed like sacrificing to a theory—like Mahomet going to the mountain, when the mountain would not come to Mahomet. It is indeed saying, with a singular accommodation, as our author quotes:

> "Alter erit tum Tiphys, et altera quæ vehat Argo

> Delectos heroas; erunt etiam altera bella;

> Atque iterum ad Trojam magnus mittetur Achilles."

The reason given why Aristotle's limitation of the copula-verb to the present tense may be justified, will also appear unsatisfactory to many of our logical readers. No doubt, as our author says, thought involves the consciousness of present mental acts; but when he adds that the office of the copula is simply to deduce the present co-existence of two objects of thought in the mind, we cannot accept of this theory without a qualification. If I put Cæsar and Pompey into relation in my mind, the relation must be in some way determined. I cannot always use the present tense without altering the predicate. If I use it in the former case, I must employ some equipollent proposition.

We regret that our limits will not allow us to go on with our analysis of Mr. Mansel's book, as we must reserve a little space for topics incidentally discussed in these pages, and always with great power of psychological analysis. Indeed, we regard the main value of the work, as a contribution to mental science, to consist in the critical skill with which a variety of questions bearing, sometimes more immediately, at other times more remotely, on logic as a science.

Our author, for example, after remarking on the severity of Cousin's criticism of Locke's definition of knowledge, as being "the perception of the agreement or disagreement of ideas," decides the point more satisfactorily than either Locke or his critics, while he, more justly than many of the latter, makes allowance for Locke's defective use of terms, owing to the unsettled state of philosophical terminology in his day. As related to *logical* judgments, we agree with Mr. Mansel that Locke's definition, in the sense he meant, is substantially correct, since, in every logical judgment, there is a certain union of "ideas" (our author would always say *concepts*), each being represented by a sign. As these ideas or "concepts" may be regarded as existing in the mind before logical predication, the logical judgment may be said to be formed by the combination of ideas or concepts. M. Cousin's objections tell only against judgments exclusively psychological. Such are all the spontaneous judgments of the mind—that is, all the actual presentations of perception and imagination, producing a realization of the presence of their objects without any logical process. Thus, *ego sum* is a primitive or psychological judgment, one to which Locke's definition will obviously not apply; for self is so presented in consciousness, that to know what we mean by *ego*, is to recognise the all-pervading sense of our own existence; so that, psychologically, the predicate and the subject are here inseparable, both in the order of nature and of time.

Our author thinks that Kant's definition of judgment is, in one respect, too narrow—in another, too wide. Kant makes thought and judgment the same, and they are both products of the understanding, which he defines the faculty of thinking or judging by means of concepts (*denken is das Erkentniss durch Begriffe.* Kritik der r. V. p. 70, *Rosenkranz*). And as Kant holds a representative theory of perception, a judgment is the representation of a representation of objects (das Urtheil is die Vorstellung einer Vorstellung desselben, i. e., eines Gegenstandes.—Ibid, p. 69). Kant expressly refuses to the intuitive faculties any function that can be called judging. It is evident that the term judgment is used with a different signification if we apply it to our mere perception of objects as present to sense or in consciousness, as compared with its logical use. In some respects, the question is one of the meaning of words; but it must be allowed to our author, that animals to whom we can hardly assign concepts which demand understanding, seem as convinced of the presence of objects as ourselves, and they, so far, judge, in the intuitive (intuitional) sense of our author. The latter thinks Kant's definition, in a logical point of view, too wide, as including all our conceptions or apprehensions; so that any object of intuition may be the subject of possible predication. We cannot further dwell on this point; but we have already remarked that we do not see how every term of a logical judgment can, *without exception*, be regarded as standing for a concept, in our author's sense of the word. At all events, our readers must see, that, laudably as he seeks to draw attention to the extraordinary merits of Kant as a suggestive writer, he by no means slavishly follows him. He justly condemns this great thinker, in a subsequent passage, for asserting that the objects of our intuition (here sensuous objects) *are not* in themselves as they appear to us; for this implies that we have a power of comparison which the hypothesis excludes. The author of the Critical philosophy here "becomes a dogmatist in negation."

There are some very instructive chapters on "Mathematical, Logical, and Psychological Necessity," of which we can only give the briefest summary. The principles of geometry are laws relating to the subjective condition of one portion of our intuitions, those which can only be presented as in space. These principles are empirical, so far as suggested in and through our experience of space; necessary as relating to the conditions under which such experience is possible to our faculties. If there exist anywhere a pair of perfectly straight lines, they cannot enclose a space. Arithmetic is founded on another internal law or condition of our mental constitution—that of time. Mathematical judgments are synthetical, in the Kantian sense, and are necessary because thought can only operate in conjunction with matter given by intuition, and intuition cannot be emancipated from its own subjective conditions. Judgments of logical necessity are analytical, and rest on the laws of thought, properly so called. They depend on the principles of identity or contradiction. Judgments of mathematical and logical necessity our author terms "judgments necessary in the first degree." They are dependent on the laws of our mental operations, and their contradictions are neither conceivable nor supposable. Judgments of psychological necessity are necessary "in the second degree." They are dependent on the restrictions of our mental constitution; and their contradictions are "supposable but not conceivable." To this class the author refers the principles of causality and substance, as examples. We wish our limits would allow of our giving a complete view of the whole dissertation respecting these two fundamental principles of the Eclectic school of France. No part of the book exhibits more to advantage the analytical power of the writer's mind, and his original talent for this kind of inquiry; which must be acknowledged by all who can follow him through the maze of conflicting theories, whatever opinion may be entertained of the questions themselves. Judgments necessary in the "third degree" are those of physical necessity, which depend on the laws of the material world, and their contradictions are supposable and conceivable, but not actually true. Finally come purely "contingent judgments," in which either contradictory may be the true or the false alternative. Thus, I am uncertain from what quarter the wind will blow to-morrow, not because this is contingent in itself, but that I am ignorant of the laws which determine meteorological phenomena, though the progress of science may raise these judgments from the category of contingency to that of physical necessity.

Discussions follow on the "matter and form of thought, on positive and negative thought, and on logic as related to other mental sciences," namely, grammar, psychology, and metaphysics. The latter term has totally altered its meaning in modern times, at least down to the period of Kant. In the Scottish schools it has been used as synonymous with empirical psychology, or what Stewart terms the "inductive philosophy of the human mind." Hence we hear of the "Scotch metaphysics." Kant distinguishes it (metaphysik) from empirical psychology, and defines it to be the science of *à priori* truth, and regards it as wholly subjective. Its ancient meaning is thus given by our author:

"Metaphysics has, from the earliest days, been distinguished as the science of being as being, in opposition to all inquiries into the phenomena exhibited by this or that class of objects.* How far such a problem is capable of solution is another question; but the mere propounding of it implies an object totally distinct from that of an inquiry into the faculties and laws of the human mind. The object of the older metaphysics has been distinguished in all ages as the one and the real, in opposition to the many and the apparent (Aristot. Metaph. iii. 2). Matter, for example, as perceived by the senses, is a combination of distinct and heterogeneous qualities. What is the *thing itself*, the subject of these qualities? Mind presents to consciousness so many distinct states and operations and feelings—what is the nature of that one mind, of which all these are so many modifications? The inquiry may be carried higher still. Can we attain to any single conception of being in general, to which both matter and mind are subordinate, and from which the essence of each may be deduced? (Wolf, Phil. Ration. Præ. § 78. Herbart *Allg. Metaph.* § 27.) Ontology, as thus explained, may be treated in two different methods, according as its exponent is a believer in τὸ ὂν or in τὰ ὄντα, in one or in many fundamental principles of things. In the former all objects whatever are regarded as phenomenal modifications of one and the same substance, or as self-determined effects of one and the same cause. The necessary result of this method is to reduce all metaphysical philosophy to a rational theology, the one substance or cause being identified with the Absolute or the Deity. According to the latter method, which professes to treat of different classes of beings independently, metaphysics will contain three co-ordinate branches of inquiry, rational cosmology, rational psychology, and rational theology. The first aims at a knowledge of the real essence, as distinguished from the phenomena of the material world; the second discusses the nature and origin, as distinguished from the faculties and affections of the human soul and of other finite spirits; the third aspires to comprehend God himself, as cognizable *à priori* in his essential nature, apart from the indirect and relative indications furnished by his works, as in natural theology, or by his word, as in revealed religion. These three objects of metaphysical inquiry, God, the world, the mind, correspond to Kant's three ideas of pure reason; and the object of his critique is to show that, in relation to all three, the attainment of a system of speculative philosophy is impossible." (p. 276.)

The notes contain some further highly important dissertations, especially on the vexed question of liberty and necessity; and, in particular, on the arguments alleged for the subjection of the human will to the law of physical necessity. The opinions of Mr. John Mill and of Sir W. Hamilton are here examined with the author's usual ability and candour.

We can only add, that we deem the work, as a whole, to be one of the most important contributions to psychological science that has yet

* This agrees with Aristotle's account of the *First Philosophy*: ἔστιν ἐπιστήμη τις ἣ θεωρεῖ τὸ ὂν. Metaph. iii. 1.

appeared. The style is, for the most part, eminently clear, the examples for illustration, generally well-chosen; and the book is well adapted accurately to inform all who can and will patiently digest it, on the true bearing of most of the great questions of speculative philosophy, and especially on the connexion between psychology and logic.

---

### Art. VI.—THE PILGRIMAGE OF THOUGHT.*

